# Molecular Analysis of the Muscle Protein Projectin in Lepidoptera

**DOI:** 10.1673/031.013.8801

**Published:** 2013-09-21

**Authors:** A. J. Ayme-Southgate, L. Turner, R. J. Southgate

**Affiliations:** 1 Department of Biology, College of Charleston, 66 George Street, Charleston, SC 29401; 2 Current address: Central Carolina Technical College, Sumter, SC

**Keywords:** alternative splicing, elastic filaments, flight muscle, insect, titin

## Abstract

Striated muscles of both vertebrates and insects contain a third filament composed of the giant proteins, namely kettin and projectin (insects) and titin (vertebrates). All three proteins have been shown to contain several domains implicated in conferring elasticity, in particular a PEVK segment. In this study, the characterization of the projectin protein in the silkmoth, *Bombyx mori* L. (Lepidoptera: Bombycidae), and the monarch butterfly, *Danaus plexippus* L. (Lepidoptera: Nymphalidae), as well as a partial characterization in the Carolina sphinx, *Manduca sexta* L. (Lepidoptera: Sphingidae), are presented. This study showed that, similar to other insects, projectin's overall modular organization was conserved, but in contrast, the PEVK region had a highly divergent sequence. The analysis of alternative splicing in the PEVK region revealed a small number of possible isoforms and the lack of a flight-muscle specific variant, both characteristics being in sharp contrast with findings from other insects. The possible correlation with difference in flight muscle stiffness and physiology between Lepidoptera and other insect orders is discussed.

## Introduction

How insect flight originated and how structures associated with flight evolved are still mostly unknown, and as groups of insects acquire additional features, they are considered “derived” compared to earlier basal groups. The flight musculature of derived insects relies on indirect flight muscles, which are known to contain connecting C-filaments providing a direct mechanical link between the muscle's sarcomeric Z-discs and the ends of the thick filaments (Trombitas 2000). In these muscles, the passive myofibrillar elasticity attributed to the C-filaments resides with several proteins, projectin, and several isoforms from the sallimus gene, including the most abundant form, which is known as kettin. Projectin, kettin, and other isoforms of sallimus are therefore proposed to be responsible for the high resting stiffness of indirect flight muscles (Granzier and Wang 1993; Moore et al. 1999; Bullard et al. 2000, 2002, 2005; Trombitas 2000; Vigoreaux et al. 2000; Kulke et al. 2001; Burkartetal. 2007).

The complete amino acid sequence of projectin is currently available in several insect species from five different orders and reveals that projectin's modular organization is highly conserved with its specific pattern of repeated motifs and unique sequences (Ayme-Southgate et al. 2008). This modular structure, as well as the arrangement of motifs, are actually common to all invertebrate projectins characterized so far, including twitchin in *Caenorhabditis elegans* and projectin in crayfish, *Procambarus clarkii*, even though the number of Ig domains at the NH_2_-terminus is lower (for example 7 rather than 8 in the crayfish; Benian et al. 1989; 1993, Oshino et al 2003).

The NH_2_-terminus of projectin contains a unique region that in previous studies was delineated into two segments: a PEVK region followed by the so-called NTCS1 segment (Nterminal conserved sequence 1; Ayme-Southgate et al. 2011). Sequence comparison of the PEVK segments across several insect species revealed a series of unique features: an enrichment in 4 specific amino acids (Proline (P), Glutamic acid (E), Valine (V), and Lysine (K)), a highly divergent primary sequence, and a complex pattern of alternative splicing (Southgate and Ayme-Southgate 2001; Ayme-Southgate et al. 2008, 2011). In all the species investigated so far, alternative splicing of the projectin PEVK region has been shown to generate isoforms ranging in lengths from 34 to 624 amino acids. and a P, E, V, and K composition from 42% to 100% (Southgate and Ayme-Southgate 2001; Ayme-Southgate et al 2008, 2011).

The vertebrate protein, titin, makes up the third, elastic filament of striated muscles. Although titin is larger in size, it contains the same domains as projectin, in particular a PEVK region, which is longer and more complex (Labeit et al. 1992; Labeit et al. 1997). The titin PEVK region undergoes extensive alternative splicing events, and variable lengths of the PEVK region found in different muscle types are associated with significant divergence in passive tension (Cazorla et al. 2000; Freiburg et al. 2000; Granzier and Labeit 2002, 2005).

Analysis of the projectin PEVK region in insects such as dragonflies, *Pachydiplax longipennis* Burmeister (Odonata: Libellulidae) and *Libellula pulchella* Drury, *Apis mellifera* L. (Hymenoptera: Apidae), and *Drosophila melanogaster* Meigen (Diptera: Drosophilidae) has shown that their flight muscles contain a short isoform that is absent from other muscles in the same insect (Ayme-Southgate et al. 2005, 2011). These same insect orders are also known to have flight muscles with relatively high stiffness (Thorson and White 1983; White 1983; Peckham et al. 1990, 1992). To evaluate a possible correlation between projectin PEVK variants and muscle stiffness, the features of the PEVK region and splicing pattern in insects with lower flight muscle stiffness need to be established. It is generally proposed that synchronous flight muscles would display lower passive tension (reviewed in Pringle 1977 and Dudley 2000). To this purpose, insects from the Lepidoptera order, which have synchronous muscles (Pringle 1981), were used. Furthermore, the Lepidoptera order was used in order to take advantage of the availability of the genomes of the silk moth, *Bombyx mori* L. (Bombycidae) (Mita et al. 2004; International Silkworm Genome Consortium 2008), and the monarch butterfly, *Danaus plexippus* L. (Nymphalidae) (Zhan et al. 2011), to establish the overall structure of their projectin genes. The sequence for NH_2_terminal region of projectin in the Carolina sphinx, *Manduca sexta* L. (Sphingidae), was determined, and the analysis of its PEVK region was completed.

The analysis showed that the overall domain pattern of the projectin protein was conserved, and the PEVK region followed the features previously identified. However, even though alternative splicing of the *M. sexta* PEVK segment occurred, the number of variants was low, and there was no evidence for the presence of a short, flight muscle-specific PEVK isoform.

## Materials and Methods

### Insects and RNA sample preparation

*B. mori* and *M. sexta* were purchased as larvae and/or pupae from Educational Science (www.educationalscience.com) and reared up to emergence of the imago, at which point they were dissected. Total RNA was purified from whole animals, isolated body parts (legs, heads, thoraces), and from flight muscles using Trizol (www.invitrogen.com) as previously described by Ayme-Southgate et al. (2008).

### Degenerate primers and splicing analysis

Degenerate primers have been described elsewhere (Ayme-Southgate et al. 2011). RTPCR reactions were performed as described before with different RNA preparations and primer sets (Southgate and Ayme-Southgate 2001). Annealing for both the RT and PCR reactions were tested using a range of temperatures to optimize each primer set. DNA fragments were isolated after agarose gel electrophoresis and subcloned into the pGEM-T easy shuttle vector (Promega, www.promega.com), which was then followed by sequencing (Genewiz Inc., www.genewiz.com). Sequences from overlapping clones were manually assembled into contigs.

### Bioinformatics analysis

For the *B. mori* projectin, contigs were isolated following tblastn searches of GenBank WGS database using a series of gene fragments from *D. melanogaster* projectin. The tblastn algorithm compares a query protein sequence to the 6-frame-translation of a DNA sequence; in this case, the contigs available for the *B. mori* genome (Altschul et al. 1990). The EST database from the two silkworm transcriptome projects, available through SILKBASE and GenBank, was also searched, and resulting EST sequences were aligned on the genomic sequence. For the *D. plexippus* projectin, a BLAST search of the recently published genome was performed using *B. mori* projectin cDNA as query. Sequence comparisons were carried out using the CLUSTALW algorithm, and the alignments were viewed in Jalview (Thompson et al. 1994, 1997).

**Supplementary Table.**
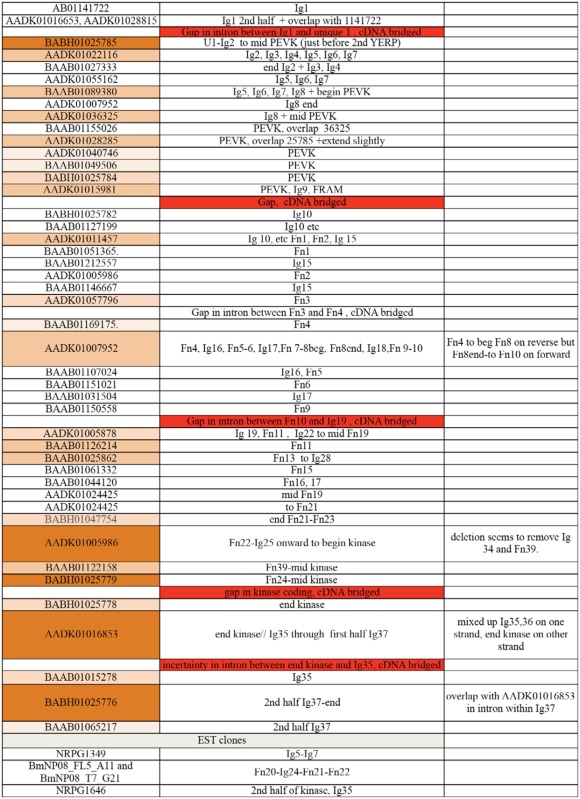
Summary of contigs from the *B. mori* genome database assembled to generate projectin genomic sequence.

## Results

### *B. mori* and *D. plexippus* projectin sequences

The annotation in the GenBank database reported a *B. mori* projectin homolog based on genome analysis of the *B. mori* Z chromosome (Koike et al. 2003). This annotation predicted a series of short proteins with names such as D-titin, kettin, and projectin-like containing immunoglobulin (Ig) and/or fibronectin type III (Fn) domains (GenBank ID: NM_001114995 for projectin-like). None of these entries represented the correct gene for the projectin protein, as they were too short and did not contain the characteristic pattern of Fn and Ig domains expected for projectin. At the start of this project, the *B. mori* projectin gene was assembled *de novo* following tblastn searches of *B. mori* supercontigs (Mita et al. 2004) available in the GenBank database using a series of peptide fragments from the *D. melanogaster* projectin. Extensive overlap was established for most of the contigs representing the *B. mori* projectin gene (Supplementary Table 1). Some of the contigs available in GenBank were probably incorrectly assembled, however, as projectin domains known to be adjacent are coded on opposite strands or in reverse order. In other cases, some contigs contained a deletion covering several domains, which were present in other contigs. Many of these issues could have resulted from the presence of repetitive sequences. Despite our efforts, five gaps remain in the genomic sequence, as repeated searches of the GenBank database fail to return contigs overlapping these gaps, and we assume that these sequences were not obtained during the original genome sequencing. We have not attempted to ‘clean’ the original assembly or to sequence the missing genomic DNA (see below).

The exon-intron pattern was predicted over most of the gene by performing translation in all three frames and visual alignment with the *D. melanogaster* projectin amino acid sequence. The PEVK region could not be entirely predicted by this approach (see below). Ambiguous splice sites were resolved by RT-PCR using *B. mori* total RNA followed by sequencing of cDNA products. In cases where the assembly of the genomic contigs was ambiguous, the prediction was verified by RTPCR amplification across the gaps/misalignment and cDNA sequencing (see Materials and Methods for details; data not shown). EST sequences from two silkworm transcriptome projects available through SILKBASE and GenBank (Mita et al. 2003) were retrieved. The few EST sequences available were consistent with the exon-intron prediction. Of the five gaps remaining in the genomic sequence, all but one occurred within intron sequences and were bridged by our own cDNA sequencing or EST data, so it is indeed a continuous gene. The one gap falling within a coding region encompassed a segment of the kinase domain. The corresponding cDNA sequence was obtained following RT-PCR, but the exact exon-intron pattern for this part of the gene is uncertain because the genomic sequence was unavailable.

The projectin gene for *D. plexippus* was retrieved from the recently available genome data (Zhan et al. 2011). The combined sequences from two super-contigs (GenBank ID # AGBW01006173.1 and AGBW01009765.1) cover the entire projectin gene, except for half of the second Ig domain, which is located in the gap between the two super-contigs and for which no genomic data are available.

**Figure 1.**
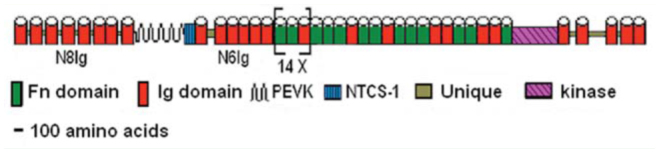
Diagram of the conserved projectin domain organization. This pattern is common to all projectin characterized so far, including the lepidopteran proteins from this study. The lg and FnIII domains are represented as barrels to reflect their globular nature. The [Fn-Fn-Ig] module is repeated 14 times within the central core region. The Nhh-terminus is composed of two tracts of eight (N8lg) and six (N6lg) Ig domains separated by the PEVK-NTCS1 region. High quality figures are available online.

**Table 1.**
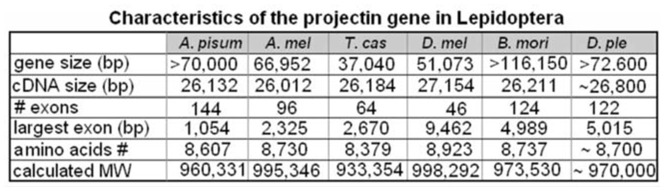
Characteristics of the projectin gene in Lepidoptera. The lepidopteran genes are compared to the projectin genes in other insect species. *Dmel: Drosophila melanogaster; Amel: Apis melllfera; Tcas: Tribolium castaneum; Api: Acyrtosiphon pisum; Bmo: Bombyx mori*, and *Dple: Danaus plexippus*.

The gene characteristics for *B. mori* and *D. plexippus* projectins are summarized in Table 1 and compared with *D. melanogaster, Tribolium castaneum* Herbst (Coleoptera: Tenebrionidae), *A. mellifera*, and *Acyrthosiphum pisum* Harris (Hemiptera:Aphididae) (Ayme-Southgate et al. 2008). The overall domain organization of projectin in the two lepidopteran insects was identical to all the projectin proteins characterized so far (Figure 1; Ayme-Southgate et al. 2008, 2011). Even though the full length of the two lepidopteran projectin genes could not be ascertained completely, they were the largest of all the projectin genes characterized, with one of the highest number of exons (Table 1). Similar to the situation found in *D. melanogaster*, the largest exon in both genes contained approximately half of the domains for the core region (the section of the protein composed of the repeated Fn-Fn-Ig modules). For the remainder of the protein, individual Ig and Fn domains were often split between two exons, a situation more similar to the one found in insects from more basal orders such as *A*, *pisum* (Ayme-Southgate et al. 2008).

### N-terminus sequence determination in *M. sexta*

To gain access to the NH_2_-terminal sequence of projectin in *M. sexta*, a series of degenerate primers based on sequence alignment of several Ig domains from the N8Ig and N6Ig tracts was used (Figure 1; Ayme-Southgate et al. 2011), as well as primers based on *B. mori* sequences. RT-PCR amplifications using *M. sexta* RNA were performed with these different primer sets, and these products were cloned and sequenced. The NH_2_-terminus region for *M. sexta* followed the standard pattern found in all projectin genes, which is two separate tracts of 8 and 6 Ig domains respectively with small interspersed linker sequences of 5 to 46 amino acids in length, separated by a unique sequence (see Figure 1).

### PEVK structure in *B. mori, D. plexippus, *and *M. sexta *genes

**Figure 2.**
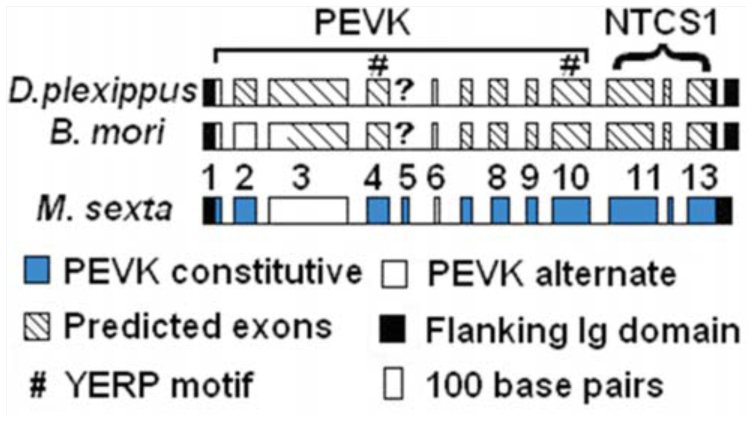
Exon-intron pattern for the PEVK-NTCS-1 segments in lepidopteran projectins. The striped boxes indicate exons that are only predicted by alignment to *Manduca sexta*. The ? indicates the exon that was not found in *Bombyx mori* and *Danaus plexippus*. PEVK exon numbers are indicated above the *M. sexta* map. Introns are not to scale. High quality figures are available online.

Species-specific primers from *M. sexta* Ig8 and Ig9 domains were used to amplify the unique sequence between the N8Ig and N6Ig regions. Internal primers were used in a second stage to try and amplify larger cDNA products for the *M. sexta* PEVK-NTCS-1 segments. The cDNA sequence was aligned to the corresponding genomic sequences in *B. mori* and *D. plexippus*. Using this approach, the PEVK and NTCS-1 exons for both insects, as well as the intron-exon boundaries in all three species, were predicted (Figure 2). Only one of the *M. sexta* exons could not be identified in either *B. mori* or *D. plexippus*, possibly because this exon was very short, with only 24 nucleotides (exon #5 in Figure 2). Several of the splice sites were confirmed in *M. sexta* through the sequencing of alternate splice products (see below).

The possibility of additional exons in the PEVK region does exist, but these exons would probably be rarely expressed, as this region was thoroughly amplified in *M. sexta*, and all resulting cDNAs were sequenced. Also, the available continuous *B. mori* and *D. plexippus* PEVK genomic sequences were translated in all three frames and visually scanned for open stretches of at least 10 amino acids with elevated PEVK content. No additional exons were predicted using this approach (data not shown).

**Figure 3.**
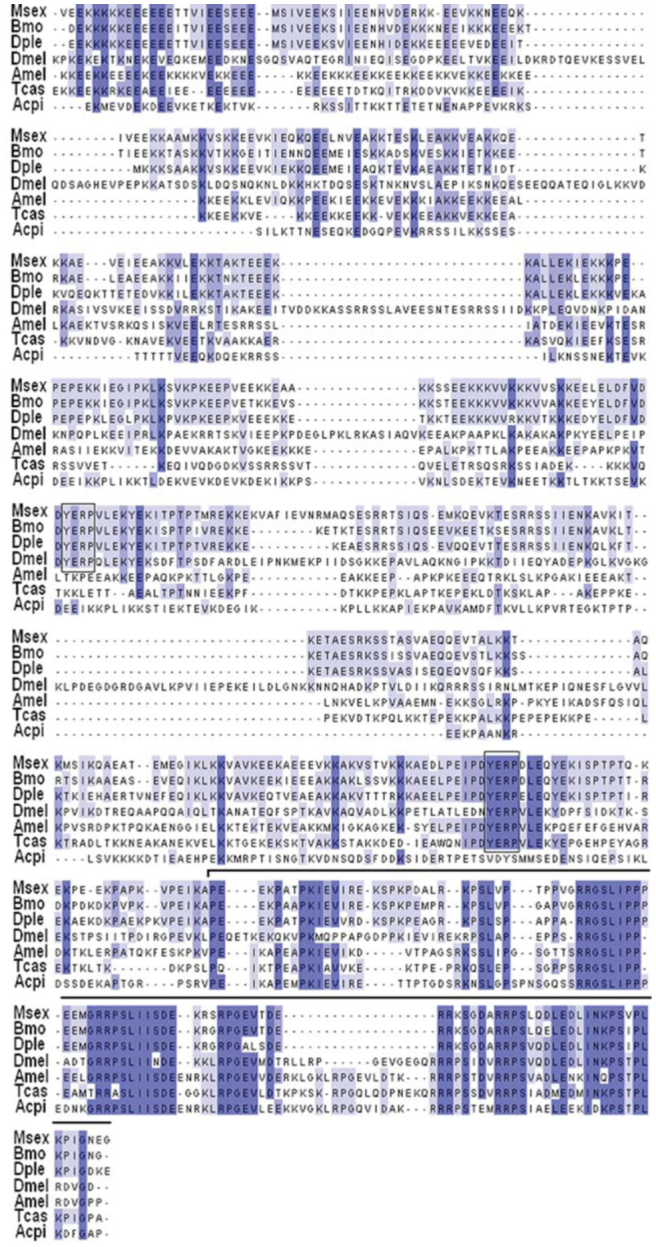
CLUSTAL-W-generated amino acid sequence alignment between PEVK-NTCS-I regions in different insects. The intensity of the shading indicates the level of amino acid identity. Black boxes indicate the position of the YERP motifs. The solid black line corresponds to the NTCS-1 segment. *Dmel: Drosophila melanogaster; Amel: Apis melllfera; Tcas: Tribolium castaneum; Api: Acyrtosliphon pisum; Bmo: Bombyx mori*, and *Dple: Danaus plexippus*. High quality figures are available online.

**Figure 4.**
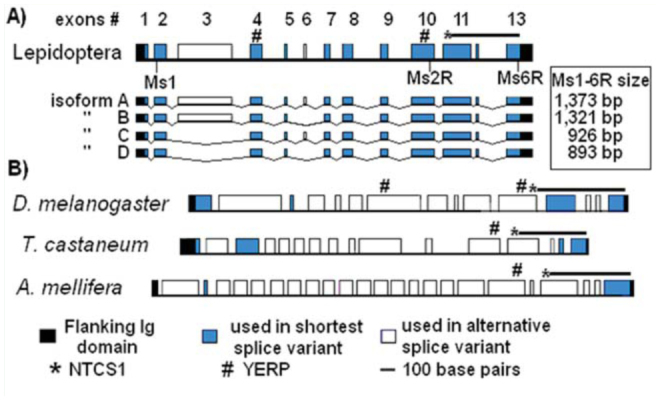
Diagram representation of the PEVK exons in different insect species. A) Exon pattern in Lepidoptera, together with the isoforms detected in *Manduca sexta*. Exon composition for the alternative variants is presented below the map together with their expected sizes following amplification with primers Ms1–6R. B) Pattern in other insects (Ayme-Southgate et al 2011). The exons used in the shortest variants are shaded in gray. Intron sizes are not to scale. High quality figures are available online.

Alignment of PEVK-NTCS-1 segments from *B. mori, D. plexippus*, and *M. sexta* with those available from some of the other species was performed using CLUSTALW and viewed with Jalview (see Materials and Methods for details). As shown by the alignment presented in Figure 3, the current subdivision of this unique sequence into two segments is supported; there is a highly divergent PEVK region and the conserved NTCS-1 segment, which is positioned just before the second stretch of six Ig domains (solid black line above alignment in Figure 3). Contrary to the PEVK segments found in other proteins (human titin, *C. elegans* TTN-1, and *Drosophila* sallimus), the projectin PEVK regions described here did not contain any repeating pattern. This was consistent with all other insect projectin PEVK segments, except for a short repeat found exclusively in *A. mellifera* projectin (Ayme-Southgate et al. 2011). The NTCS-I region was described as the largest conserved region when comparing projectins from basal (dragonfly) and more derived insects (Ayme-Southgate et al. 2011). In derived insects, including the three lepidopteran sequences described here, this conserved segment was slightly longer (by 28–35 amino acids) and began with a ‘YERP’ motif (boxed residues in the alignment; Figure 3). This conserved block was not present in the sequences of *Pediculus humanus* L. (Phthiraptera: Pediculidae), *A. pisum*, or the two dragonfly species, *P. longipennis* and *L. pulchella* (Ayme-Southgate et al. 2011). Two YERP motifs were present in the lepidopteran and *D. melanogaster* PEVK regions (black boxes in Figure 3 and # in Figure 4).

### Alternative isoforms of the PEVK-NTCS-1 segments in different muscle types

The alternative splicing pattern for the PEVKNTCS-1 region was also ascertained, and the analysis indicated that the PEVK region was the site of several alternative splicing combinations. As shown in Figure 4, there were only two exons that could be alternatively spliced to generate the shortest form (exons # 3 and 6), compared to 11 exons in *D. melanogaster* and *T. castaneum* and up to 21 in *A. mellifera*. Only 4 PEVK variants were detected in *M. sexta* PEVK, compared to at least 10 in *D. melanogaster* (Southgate and Ayme-Southgate 2001). The longest splice variant identified in *M. sexta* would encode a 377 amino acid-long PEVK region, and the shortest form would be 205 amino acids. The length difference between the longest and shortest variants was therefore not as ‘striking’ in *M. sexta* as it is in other insects, for example 75 and 530 amino acids for the shortest and longest variants respectively in *D. melanogaster* (Table 2). Also, the YERP motifs were included in all of the *M. sexta* PEVK variants, whereas they were excluded from the short variant in other insects (Figure 4). The characteristics of the PEVK and NTCS-1 segments for all three lepidopteran genes are summarized in Table 2 together with the corresponding regions in other insect projectin proteins.

**Table 2.**
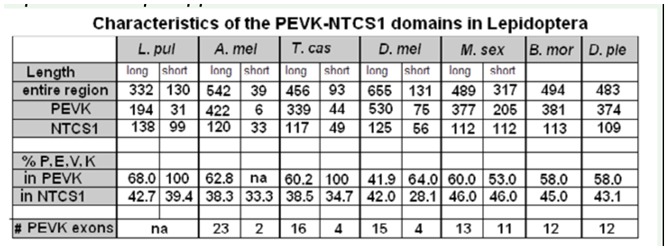
Characteristics of the PEVK-NTCS-1 regions in Lepidoptera as compared to other insects. *L.pul: Libellula pulchella, A.mel:* *Apis mellifera; T.cas: Tribolium castaneum; D.mel: Drosophila melanogaster; M.sex: Manduca sexta; B.mor: Bombyx mori;* and *D.ple: Danaus plexippus*.

The long form of the PEVK segment in all lepidopteran genes was most similar in length and PEVK content to the *T. castaneum* sequence. In contrast, the PEVK content of the short form, but not its length, was closer to the short variant of *D. melanogaster*. The short isoform in *M. sexta* also had a lower P, E, V, and K content than the longest isoform; this was in sharp contrast to the situation in all other studied insects, where the P, E, V, and K, content of the short variant can be as high as 100 percent (see Table 2).

The presence and specificity of alternative isoforms in the PEVK-NTCS-1 region were ascertained by performing RT-PCR amplification using RNAs extracted from several body parts, as well as from isolated flight muscles from *M. sexta* (see Materials and Methods for details). Primers were designed from exons flanking and internal to the PEVK-NTCS-1 segments.

**Figure 5.**
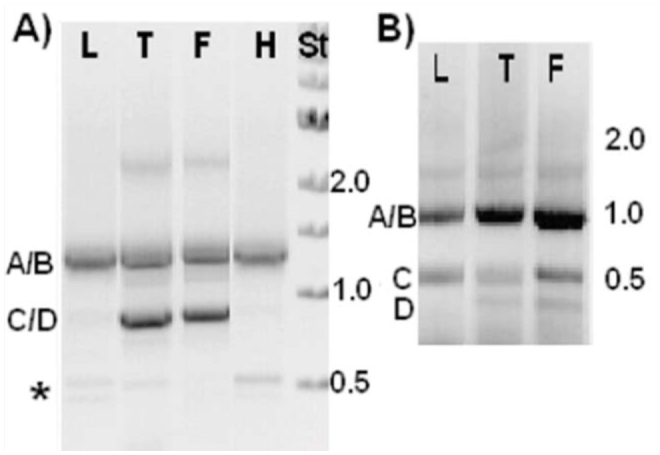
The PEVK variants are identical in all muscle types in *Manduca sexta*. Products of RT-PCR reactions were analyzed by gel electrophoresis. A): primers Ms1–6R and B): primers Msl2R (see Figure 4). The muscle types are as follows: L: leg, T: whole thorax, F: flight muscle, and H: head. St: I kb ladder from New England Biolabs (www.neb.com). Sizes are indicated on the right. A/B and C/D refer to the isoforms depicted in Figure 4. * is a contamination product corresponding to *M. sexta* hemocytin gene as identified by sequencing and BLAST homology search. High quality figures are available online.

The variant composition was qualitatively identical across muscle types irrespective of the primer pair used for the amplification. As presented in Figure 5A in the Ms1–6R reaction, the isoforms A and B (1,373 and 1,321 bp respectively; see Figure 4) were present in all muscles, and the C/D isoforms (926 and 893 bp respectively) were present in thorax and flight samples, as well as faintly in leg and head samples. The Ms1-6R primer set also faintly amplified two products around 500 bp, which were present in all muscle types. These products were sequenced and correspond to *M. sexta* hemocytin gene as identified by a BLAST homology search (Kotani et al. 1995; Tanaka et al. 2008). The reason for this reproducible amplification is unknown.

The Ms1-6R primer set amplified the entire PEVK-NTCS-1 region. Because short PCR products are favored in PCR amplification reactions, the absence of projectin products shorter than 800 bp in the Ms1–6R reaction was unlikely due to the low abundance of any short isoform. For the same reason, relative contribution of short and longer PEVK variants could not be ascertained completely from these data, even though isoforms A/B seemed to be the most abundant variants in both head and leg RNA samples.

Isoforms A, B, and C were also detected in all muscle types using primers Ms1–2R (Figure 5B). The shortest 480 bp (corresponding to isoform D) product in the Ms1–2R reaction was not detectable in the leg RNA sample. This product only differed from isoform C (corresponding to the 500 bp product) by the exclusion of exon 6 (see Figure 4), which wasonly 33 bp in length. So, isoform D could be considered a flight muscle-specific isoform.

## Discussion

In this study, further evidence for the organization of the NH_2_-terminal region of projectin into two tracts of 8 and 6 Ig domains separated by a unique sequence is provided. This unique region of the protein can be further divided into two segments. One segment showed little to no sequence conservation *per se*, but displayed biased amino acid content with predominantly P, E, V, and K residues and is considered the ‘true’ PEVK region. In contrast, the second segment, found just before the second stretch of six Ig domains, was a highly conserved sequence of 112–138 residues in length and has been named NTCS-1. The importance of this region for positioning over the length of the sarcomere and/or posttranslational modifications is unknown at this time. BLASTp search of the reference protein database yielded no significant homology other than projectin proteins.

The potential for alternative splicing in *M. sexta* muscles is limited, with only 2 alternatively spliced exons identified and 4 possible variant combinations. This is in contrast to 11 exons in *D. melanogaster* and *T. castaneum* and 21 in *A. mellifera*. In *M. sexta*, the length of the shortest PEVK variant (isoform D) represented 54% of the longest variant. In contrast, the same ratio varied from approximately 15% in dragonflies, *T. castaneum*, and *D. melanogaster* to less than 2% in *A. mellifera*. Therefore, the shortest isoform was not considerably shorter than the long isoform in the flight muscle of *M. sexta*. The shortest isoform (D) in *M. sexta* also had a lower P, E, V, and K content than the longest isoform (A); this was in sharp contrast to the situation in all other studied insects, where the short variant can be as high as 100% P, E, V, and K (see Table 2).

Isoform D was the only splice variant specific for flight muscle, and it differed from isoform C by the exclusion of only 11 amino acids encoded by exon #6. So, even though isoform D could be considered a flight muscle-specific isoform, it was not very different from the other isoforms, which were present in all muscle types. This is in contrast with previous observations carried out in other insects, namely that the flight-muscle-specific isoform is strikingly shorter than any other PEVK variants (Ayme-Southgate et al. 2004, 2011).

In vertebrates, the sarcomeric passive tension of striated muscles have been correlated with the size and composition of titin's extensible regions, in particular its PEVK segments (for example Cazorla et al. 2000; Freiburg et al. 2000; Trombitas et al. 2000; Granzier and Labeit 2005; Granzier et al. 2007). In the titin model, for a given sarcomere length, shorter PEVK segments lead to a high resting tension, whereas a longer extensible region results in a lower force (reviewed in Granzier and Labeit 2005).

In derived insects with asynchronous flight muscle, the C filaments composed of kettin/sallimus and projectin have been shown to be a source of the myofibrillar stiffness in flight muscles (Moore et al. 1999; Bullard et al. 2000; Hakeda et al. 2000; Kulke et al. 2001; Bullard et al. 2006). In these indirect flight muscles, according to the titin model, projectin molecules with a short PEVK region would contribute to their elevated stiffness. On the other hand, it is generally proposed that synchronous flight muscles, such as those of lepidopteran, have higher muscle strain and lower passive tension (reviewed in Dudley 2000). Additional studies of other insects with synchronous flight muscles will be required before a precise correlation can be established, but the current study leads us to propose that, by analogy to the spring model described for titin, long PEVK variants will be associated with muscles with low passive stiffness, higher strain, and synchronous physiology, whereas a short PEVK sequence would contribute to high myofibrillar passive stiffness.

## Abbreviations:

Fn,: fibronectin type III
Ig,: immunoglobulin
NTCS-1,: N-terminal conserved sequence 1
